# Preoccupation with Body Weight and Under-Reporting of Energy Intake in Female Japanese Nutrition Students

**DOI:** 10.3390/nu12030830

**Published:** 2020-03-20

**Authors:** Masaharu Kagawa, Andrew P. Hills

**Affiliations:** 1 Institute of Nutrition Sciences, Kagawa Nutrition University, Saitama 350-0288, Japan; 2 School of Public Health, Curtin University, Bentley, WA 6845, Australia; 3 Institute of Health and Biomedical Innovation, School of Exercise and Nutrition Sciences, Queensland University of Technology, Brisbane, QLD 4059, Australia; andrew.hills@utas.edu.au; 4 Faculty of Public Health, Mahidol University, Bangkok 10400, Thailand; 5 School of Health Sciences, College of Health and Medicine, University of Tasmania, Hobart, TAS 7250, Australia; 6 Mater Research Institute, The University of Queensland, South Brisbane, QLD 4101, Australia

**Keywords:** under-reporting, energy intake, body image, Japanese females, dietary assessment

## Abstract

The present study aimed to examine associations between body image and under-reporting in female Japanese university students enrolled in a nutrition degree program. A total of 100 participants (aged 18–29 years) completed (1) a self-administered questionnaire including the Ben-Tovim Walker Body Attitudes Questionnaire (BAQ), (2) a dietary assessment using a brief-type self-administered diet history questionnaire (BDHQ), (3) a physical activity assessment using Bouchard’s Physical Activity Record (BAR) and a tri-axial accelerometer, (4) detailed anthropometry, and (5) body composition assessment. Based on the energy intake to basal metabolic rate ratio (EI:BMR) and using a cut-off point of 1.35, 67% of participants were considered under-reporters (URs). While there was no between-group difference in BMI, URs had significantly (*p* < 0.05) greater percentage body fat (%BF) and trunk fat (%TF) compared with non-URs. Regression analyses indicated accuracy of body perception and a discrepancy between current and ideal weight were associated with EI:BMR, whereas the salience subscale of the BAQ was associated with reported EI. The study raises concerns regarding the validity of EI reported from young Japanese females as they are known to have a strong preoccupation with thinness, even with an acceptable BMI and health and nutritional knowledge.

## 1. Introduction

Accurate assessment of one’s energy intake (EI) and energy expenditure (EE) is crucial for an understanding of energy balance (EB) and associated health problems, including dietary insufficiency and overweight/obesity. Dietary assessment provides information on both quality and quantity of intake. However, regardless of the technique(s) used (e.g., 24-h recall, food frequency questionnaire [FFQ], or weighed food records), dietary assessments have a common limitation of over- or under-reporting. Whether unconscious or intentional, misreporting will affect both estimation of energy and nutrient intake and may lead to the provision of misleading advice.

Earlier research suggests that females are more likely to under-report food intake than males [1] and larger individuals, usually expressed as body mass index (BMI: kg/m^2^), have a greater tendency to under-report [2,3,4]. Previous studies have also suggested the possible influence of body image [2,4], defined as the subjective image towards one’s body, irrespective of their actual appearance [5]. This finding suggests that individuals with an acceptable body mass or body composition (e.g., percentage body fat [%BF]), may still be at risk of under-reporting EI due to a distorted body image.

Japanese females are known to have a strong desire for thinness [6] and have higher body dissatisfaction and large discrepancies between current and ideal weight or physique as determined by BMI, compared with other ethnic groups [7,8]. In recent decades, a large proportion of young Japanese females have been classified as “underweight” (BMI below 18.5 kg/m^2^) [6] and a recent National Health and Nutrition Survey in Japan (NHNS-J) reported that 21.7% of females in their 20′s were underweight in 2017 [9]. On the other hand, young Japanese females are reported to have an accurate perception of their current weight [10,11] while females more generally, under-estimate their weight [12]. However, studies in which objective physical measurements were taken have suggested that young Japanese females are unable to justify their heaviness [11] or fatness [11,13] when compared with existing health definitions [14,15]. These findings suggest that young Japanese females within the “healthy” weight range report body dissatisfaction if their weight is outside of the range which they perceive as “acceptable”. Therefore, it may be hypothesized that young Japanese females have a high likelihood of under-reporting EI, despite having a “healthy” body mass or BMI. However, no study to date has examined the association between body image and under-reporting in this population. 

While body image may influence actual eating behavior and energy intake as well as reporting of the same, lifestyle may associate with nutritional knowledge of individuals. Few studies have examined EI and EE of university students enrolled in health and/or nutrition degrees [16,17,18]. A study by Mealha and colleagues [17] indicated that nutrition students reported less fat consumption and health (nutrition and other health degrees) students engaged in more physical activity of higher intensity than non-health students. Further, an Australian study reported that female undergraduate nutrition students with high cognitive restraint had greater exercise EE that may lead to a low energy availability [18]. However, these studies were small in sample size [17,18] and did not explore the influence of body image on the likelihood of under-reporting EI. The present study therefore, aimed to examine associations between body image and under-reporting in young Japanese female university students enrolled in a nutrition degree.

## 2. Materials and Methods 

### 2.1. Participants

Japanese females enrolled in a Nutrition Sciences program at a private university in Japan were invited to participate in the study via flyers on noticeboards around the campus. Eligibility criteria included being female and aged between 18 and 30 years, self-identification as Japanese, without health problems that affect daily living (e.g., type II diabetes mellitus, kidney disease, osteoporosis, Down syndrome) or injuries (e.g., fractures, paralysis), not under medical prescription for serious health conditions or enrolled in a weight loss program, and not being pregnant.

### 2.2. Ethical Approval and Consent

All participants were given an information package, confidentiality of data was explained, along with the right to withdraw from the project at any time without any adverse consequence. Participants signed a written informed consent form prior to participation in the study and were provided with an incentive of ¥5000 upon their successful completion of all components of the study. A total of 109 participants were recruited. Of these, 105 participants signed a consent form and participated in the study but two did not complete all assessments and three did not meet all inclusion criteria. As a result, a total of 100 participants were included in the current analysis with a drop-out rate of 1.9%. The majority (72%) of participants were first year students. The study was approved by the Human Research Ethics Committee of Kagawa Nutrition University (approval number 72).

### 2.3. Self-Administered Questionnaire

A questionnaire booklet with questions on demographics, living conditions, lifestyle and body image was distributed to each participant. Examples of lifestyle questions included perceived health status, effort to maintain body weight, past experience of weight management, frequency of meals and regularity of exercise. Participants were instructed to complete the questions prior to anthropometry and body composition assessments to avoid bias. The booklet was collected and checked by the researcher in a face-to-face setting to check for any missing information and for confirmation of responses. 

#### 2.3.1. Body Satisfaction Scale (BSS)

The Body Satisfaction Scale (BSS) is used to assess body satisfaction/dissatisfaction of 16 body parts [19]–eight from the head (head, face, jaw, teeth, nose, mouth, eyes, and ears) and eight from the body (shoulders, neck, chest, tummy, arms, hands, legs, and feet). Participants were asked to rate each body part from (1) very satisfied to (7) very unsatisfied. The higher the score the greater the dissatisfaction for the particular body part. The scale provides three summative scores: the sum of all body parts provides a “general” body dissatisfaction (BSS_General_) score whereas “head” dissatisfaction (BSS_Head_), and “body” dissatisfaction (BSS_Body_) can be determined from the total score regarding body parts of each region. While Slade [19] reported that “ears” and “neck” had negligible loadings and therefore can be omitted from the calculation of BSS_Head_ and BSS_Body_ scores, the present study included both variables to follow the same protocol of a Japanese translated version [20]. The Cronbach’s alpha of the scale in this study was 0.82.

#### 2.3.2. Ben-Tovim Walker Body Attitudes Questionnaire (BAQ)

The Ben-Tovim Walker Body Attitudes Questionnaire (BAQ) [21] consists of 44-items to assess a broad range of attitudes that individuals hold towards their bodies. Items were rated on a five-point Likert scale that ranged from (1) strongly disagree, to (5) strongly agree and therefore the total score has a range of 44–220. The BAQ can provide total score (BAQ_Total_) as well as subscales for the following: (1) Feeling fat (BAQ_Fat_), (2) Body disparagement (BAQ_Disparagement_), (3) Strength and fitness (BAQ_Strength_), (4) Salience of weight and shape (BAQ_Salience_), (5) Attractiveness (BAQ_Attractive_), and (6) Lower body fatness (BAQ_Lowerbody_). Results from 504 female respondents showed a high internal-consistency of 0.87, a high correlation coefficient of 0.92, satisfactory test–retest reliability for the total score (r = 0.83) and for each subscale, and good convergent validity with existing instruments [21]. For the present study, a Japanese-translated version was utilized [22] and the Cronbach’s alpha was 0.85.

#### 2.3.3. Dietary Assessment

Participants were instructed to complete a brief-type self-administered diet history questionnaire (BDHQ, DHQ Support Centre, Japan) [23] to assess EI. The BDHQ is a food frequency questionnaire that estimates energy and nutrient intake based on food intake over the past month. It consists of 80 questions on a range of food groups including meat and meat products, seafood, eggs, bean products, vegetables including seaweeds and mushrooms, fruits, confectionery, noodles, a range of beverages including alcohol. The instrument also questions the frequency and amount of rice and miso soup consumed as well as use of seasonings including soy sauce. The instrument has been commonly utilized in Japan and it has been validated in a wide range of populations including healthy adults [24,25], pregnant women [26] and also in the elderly [27]. Explanation before the administration of the questionnaire, along with a checking process, was conducted by a registered public health nutritionist in a face-to-face setting. Dietary analysis was conducted by an external company (DHQ Support Centre, Japan) and provides details of intake of total energy as well as major macro- and micro-nutrients. EI, along with energy contributions from protein (%E_Protein_), carbohydrate (%E_CHO_), and fat (%E_Fat_) determined from dietary analysis, were used in statistical analyses.

#### 2.3.4. Physical Activity Record

To determine daily EE, Bouchard’s Physical Activity Record (BAR) [28] was utilized. The BAR is a self-administered diary that records intensity and duration of physical activity across 15-min intervals for three days. Participants were instructed to record their activities for three consecutive days including at least one weekend day. All activities were rated on a scale of 1 (sedentary) to 9 (intense manual labour or high-intensity sports) and total daily EE can be calculated by summing total time spent in activities of different intensity. An average of estimated EEs from recorded days was utilized for analysis (EE_Record_).

### 2.4. Tri-Axial Accelerometer

Participants were instructed to wear a tri-axial accelerometer (EW-NK52, Panasonic Corp., Japan) as they recorded the BAR. The accelerometer is 3.57 cm × 7.5 cm × 1.54 cm and weighs approximately 36 g, including the battery. All participants carried the accelerometer in a waist mounted pouch from the time they woke up until bedtime, except activities where the device may be immersed in water (e.g., during showering and swimming). Daily step counts and energy expenditures (EE_Acc_) were obtained from the device and the average of these variables was utilized in analyses. The validity of the device to estimate light-intensity physical activity has been reported in a study with older adults [29]. 

### 2.5. Anthropometry

All participants were assessed using a detailed anthropometric protocol consisting of 43 variables (stature, body mass, sitting height, arm span, eight skinfolds, 14 girths, eight lengths, and nine breadths). All measurements, except abdominal circumference, were conducted according to the protocol of the International Society for the Advancement of Kinanthropometry (ISAK) [30]. Abdominal circumference was measured at the level of the umbilicus based on the protocol to assess metabolic syndrome in Japan [31]. Stature and sitting height were measured using a digital stadiometer (AD-6227, A&D company Ltd., Japan) to the nearest 0.1 cm and body mass was measured using a multi-frequency bioelectrical impedance analysis (MFBIA) device (Innerscan Dual RD-800, Tanita Corp., Japan) to the nearest 0.1 kg. Arm span was measured using wall-mounted graph paper to the nearest 0.1 cm. Skinfolds were measured using a Harpenden skinfold caliper (British Indicators Ltd., England) to the nearest 0.1 mm. Girths were measured using a steel tape (W606PM, Lufkin, the United States) whereas bone lengths and breadths were measured using a segmometer, a large sliding caliper and a small sliding caliper (Rosscraft Innovations Inc., Canada) to the nearest 0.1 cm. All measurements were taken by a Level three anthropometrist accredited by ISAK with an acceptable level of intra- and inter-tester technical error of measurements (TEM) [32,33]. All participants were instructed to wear light clothes with no socks and shoes. Participants were also informed to avoid consuming food and refrain from exercise as well as to void their bowels prior to the measurement session in order to maintain normal hydration status. BMI, sum of eight skinfolds (Σ8SF), abdominal-to-height ratio (AHtR), and waist-to-hip ratio (WHR), were calculated from the measurements. To determine the distribution of body size, the BMI of participants were subdivided according to the WHO and the Japan Society for the Study of Obesity (JASSO) cut-off points, plus public health action points considered appropriate to reflect body fat accumulation for Asians (i.e., <18.5 kg/m^2^, 18.5–22.9 kg/m^2^, 23.0–24.9 kg/m^2^ and ≥25.0 kg/m^2^) [14,31,34]. 

### 2.6. Body Composition Assessment

Body composition was assessed using a MFBIA device (Innerscan Dual RD-800, Tanita Corp., Japan) [35]. After removing all jewelry and metal then wiping the surface of both hands and feet, participants were instructed to stand on the device maintaining an upright posture. Percentage body fat (%BF), percentage trunk fat (%TF), and visceral fat level were estimated from the device and utilized for analysis.

### 2.7. Statistical Analysis

To determine under-reporters, the basal metabolic rate (BMR) of participants was calculated using an equation proposed by Ganpule and colleagues [36] for healthy adults (> 20 years). The estimated energy requirement (EER) of participants was then determined using a physical activity level (PAL) of 1.75, as suggested in the Dietary Reference Intake for Japanese females of 18–29 years [37]. EB was determined from the differences between EI, analyzed from the BDHQ and EER, as well as EE_Record_ and EE_Acc_. Suspected under-reporters (URs) were then determined based on a ratio between EI and BMR (i.e., EI:BMR). Goldberg and Black [38] stated that it is very unlikely for healthy adults to have a habitual PAL lower than 1.35. Therefore, participants with a EI:BMR below 1.35 were considered to be URs. 

Normality of data was examined using the Shapiro–Wilk test and normal Q-Q plots. Anthropometric, body composition and body image variables were compared between URs and non-URs using independent t-test. Variables that did not show normal distribution were examined using the Mann-Whitney test. In addition, regression analyses were conducted to determine variables that associated with EI:BMR and EI. Variables utilized in the analyses included demographic (age, grade), physical (stature, body mass, BMI, %BF, %TF, visceral fat level, WHR, AHtR, Σ8SF, BMR, EER), lifestyle (EI, energy contributions from protein, carbohydrate and fat, participation to a regular exercise, EE_Record_, EE_Acc_, step counts), and body image (perceived current weight, perceived ideal weights, difference between measured body mass and current weight [Diff_measured–current_], difference between measured body mass and perceived ideal weight [Diff_measured–ideal_], BSS scores for each body part, BSS_General_, BSS_Head_, BSS_Body_, BAQ_Total_, BAQ_Fat_, BAQ_Disparagement_, BAQ_Strength_, BAQ_Salience_, BAQ_Attractive_, and BAQ_Lowerbody_). All statistical analyses were conducted using the IBM SPSS Statistics package (version 24.0, IBM, Chicago, IL, USA). Results are shown as mean ± standard deviation (SD) and all statistical tests used a significance level of 0.05, unless otherwise stated.

## 3. Results

Demographic characteristics are shown in Table 1. Age, stature, and body mass of the participants were 19.1 ± 1.4 years, 158.9 ± 5.2 cm and 53.0 ± 6.6 kg. Indices for obesity such as BMI, Σ8SF, AHtR, %BF, %TF, and visceral fat level were 21.0 ± 2.3 kg/m^2^, 127.3 ± 38.4 mm, 0.47 ± 0.05, 28.1 ± 4.5%, 27.2 ± 5.4%, and 2.4 ± 1.7, respectively. The BMI distribution of the participants for categories <18.5 kg/m^2^, 18.5–22.9 kg/m^2^, 23.0–24.9 kg/m^2^ and ≥25.0 kg/m^2^ was 10.0%, 73.0%, 13.0%, and 4.0%, respectively. From the questionnaires, more than 63% of participants perceived themselves as “unhealthy” and approximately the same proportion reported they had experienced weight management in the past. While the vast majority (85%) reported having three meals every day, 47% reported no engagement with regular exercise of 30 min or more per session during a week and only 29% participated in regular exercise at least twice a week.

The average total EI of participants was 1523 ± 457 kcal (Table 2) however, considerable individual variability was evident ranging between 536 and 2735 kcal. Although not reaching statistical significance, total EI was negatively correlated with body mass, BMI, Σ8SF, as well as %BF, %TF, and visceral fat level (r ranged between −0.108 and −0.196). Total EI from the BDHQ was significantly (*p* < 0.01) lower than estimated EER as well as EE from the BAR and accelerometer. A comparison between EER and EE_Record_ showed an average of −73 ± 247 kcal (minimum: −1147 kcal, maximum: 443 kcal). A difference between EER and EE_Acc_ (i.e., EER–EE_Acc_) on the other hand, showed an average of 324 ± 171 kcal (minimum: −53 kcal, maximum: 1048 kcal) (data not shown). Depending on the method used to estimate EE, 75–87% of participants showed a negative energy balance. Based on the average EI:BMR, 67% of participants had a EI:BMR below 1.35 and were therefore classified as suspected URs.

As the results indicated the presence of suspected URs, participants were grouped into suspected URs and non-URs based on the EI:BMR cut-off point of 1.35. There were no significant differences in distribution of grades between the groups. Although body mass was significantly different between the groups (*p* < 0.05), no significant between-group difference in age, stature, and BMI was observed (Table 3). Similarly, there were no significant differences in distribution of participants across BMI categories in each group. However, all participants with a BMI greater than 25 kg/m^2^ and 76.9% (10/13) of the participants with BMI between 23.0–24.9 kg/m^2^, were classified into the UR group (data not shown). While there were no differences in body size between groups, the UR group had significantly greater %BF, %TF and visceral fat level (all *p* < 0.05). These results suggest that, on average, the suspected URs had considerably greater fat accumulation than their counterparts for a similar body size. With regard to lifestyle, there were no differences in eating pattern (e.g., frequency of eating three meals a day, frequency of skipping breakfast, awareness of the content of meals, and eating speed) and also participation in regular exercise (data not shown).

The UR group showed significantly (*p* < 0.05) greater BMR and EER compared with the non-UR group (Table 4). Comparison of EE from different methods showed that the EE_Record_ was slightly but significantly (*p* < 0.05) greater among the URs while EE_Acc_ and step counts were comparable. On the other hand, the UR group reported significantly (*p* < 0.01) lower EI than their non-UR counterparts although their energy contribution from major nutrients (carbohydrate, protein, and fat) were similar.

For variables related to body image, both groups perceived their current weight accurately (the URs: 53.7 ± 6.8 kg; the non-URs: 51.1 ± 4.7 kg) with only 0.23 ± 1.2 kg difference between measured and self-reported weight for the URs and 0.09 ± 0.72 kg for the non-URs. The results also showed comparable ideal weight for height between the groups. The difference between measured body mass and perceived ideal weight was only 2 kg greater for the URs and not statistically significant. As shown in Table 5, scales utilized to examine body dissatisfaction and body concern did not differ between groups, except for the BSS score for the abdominal region (*p* < 0.05. Data not shown).

In order to determine variables that associate with EI:BMR, stepwise multiple regression analyses were conducted. When comprehensive variables examined in the present study were included, EI and EER, as well as accuracy in body perception (i.e., difference between measured body mass and perceived current weight), and a discrepancy with perceived ideal weight and body dissatisfaction of specific body parts, were associated with the EI:BMR (Table 6). Since EI was included in the derived equation, factors that influence EI were further explored. The results showed that only the BAQ salience to weight and shape subscale score was associated with EI with R^2^_adj_ of 0.112.

## 4. Discussion

The present study assessed the influence of body image on under-reporting among young female Japanese university students whose average stature (158.9 ± 5.2 cm) and body mass (53.0 ± 6.6 kg) were comparable to values reported in a recent NHNS-J for 19-year-old females (stature: 158.0 ± 5.4 cm, body mass: 52.2 ± 7.0 kg) [9]. However, there was significant individual variability in some measures (e.g., more than 20 cm for stature and more than 40 kg for body mass). In the present study, more than 60% of participants perceived themselves as “unhealthy” despite their average BMI and %BF being within the normal range [14,15]. Previous studies have suggested that young Japanese females are likely to justify their level of fatness via weight relative to their height [13] but not necessarily have a correct understanding of “heaviness” or “fatness” when compared with existing classifications for health [11]. Considering the relatively large proportion of participants who reported efforts to maintain or achieve their ideal weight and reported undertaking weight management in the past, it may be possible that some perceived themselves as “unhealthy” based on inappropriate standard for their weight.

In addition, it may possible that an unhealthy lifestyle, such as low engagement with regular exercise (47%), may have influenced their response. The observed result was consistent with outcomes of an earlier study by Korn and colleagues that reported 47.2% of female nutrition students did not engage in any physical exercise [16]. In addition, while the recent NHNS-J reported that only 28.6% of Japanese females in their 20′s regularly participates in exercise for at least 30 min twice a week [9], a comparable proportion of participants reported their participation in regular exercise (29%). This suggests that Japanese female students enrolled in a nutrition degree program may not necessarily increase their frequency of exercise when compared with females of the same age group in general. However, considering the fact that participants have health and nutrition knowledge including the physical activity recommendation, participants might have recognized their unhealthy lifestyle more than those without such knowledge and therefore negatively impacted the perception of their own health status.

Depending on the method, 75–86% of participants showed a negative EB. A number of studies have reported that dietary assessment methods, including the FFQ, tend to show EI lower than total EE determined from the doubly labeled water (DLW) technique [39]. The proportion of participants with a negative EB was higher than reported in a study of female university students enrolled in nutrition degree in Australia (35.0%) [18]. The difference may be largely due to methodology however another possible factor may be ethnic differences in body image.

Since a negative EB in this population could be due to a combination of under-reporting and also low EI or excessive EE associated with weight loss behaviours, the study aimed to determine suspected URs using EI:BMR. Based on the cut-off point of 1.35 for EI:BMR as proposed by Goldberg and colleagues [40], 67% of participants were considered suspected URs and not reporting their habitual dietary intake. Because of the comparable distribution of grades between the groups, it can be assumed that nutritional knowledge did not influence EI:BMR. This also suggests that young females in the general public may have a risk of under-reporting comparable to the study population. Considering the small difference in BMR between groups (40 kcal), it can be assumed that a major factor that contributed to the difference in EI:BMR was EI. Previous studies have reported that those who under-estimate EI are heavier than those who don’t [2,3,4]. The present study showed a very weak negative correlation between EI and variables for body mass or adiposity. This may suggest the possibility that some young Japanese females with high body mass or %BF report a low EI. The study also showed a significant between-group difference in body mass, finding consistent with previous studies. However, there were no significant differences in BMI suggesting that body size may not increase the likelihood of under-reporting. In addition, while the URs had greater fat accumulation, the level of fat accumulation was still within the acceptable range to achieve optimal health (e.g., 18.0–30.0% as suggested by Wilmore et al. [15]). Therefore, under-reporting by young Japanese females may occur regardless of weight status and not necessarily associated with excessive adiposity. Regression analyses showed that body image components such as accuracy in perceiving current weight and magnitude of discrepancy between actual body mass and perceived ideal weight may strongly influence EI:BMR of young Japanese females.

We examined body image from three different perspectives–body perception, body dissatisfaction, and body concern. Participants showed relatively accurate perception of current weight, less than 250 g on average regardless of group, a finding consistent with a previous study [11]. Therefore, unlike females living in other countries [12], the present study confirmed a high reliability of self-reported current weight by young Japanese females. Although a difference between measured body mass and perceived current weight was found to be associated with EI:BMR, it may not strongly influence many young Japanese females. The study also showed no group differences in body dissatisfaction as measured by the BSS_General_ and subscales except for the abdominal region. Scores were comparable with previous findings from 191 Japanese female university students (BSS_General_ score: 73.4 ± 11.2, BSS_Body_ score: 38.4 ± 7.3) [20] and higher than the original study by Slade [19], suggesting young Japanese females are likely to have higher body dissatisfaction than other ethnic groups in general. While previous studies have suggested an association between body dissatisfaction and under-reporting of EI [2,4], our results may indicate that the influence of body dissatisfaction on EI and resultant EI:BMR may be limited compared with other ethnic populations.

The only variable associated with EI was the salience subscale score of the BAQ. This may suggest that participants with strong preoccupation towards weight and shape may alter EI, either actual intake or the way they record it. In the present study, the majority of participants had a BMI within the acceptable range according to the WHO or JASSO [14,31]. However, the UR consisted of a greater proportion of individuals with a BMI above 23 kg/m^2^ and likely to have a %BF greater than 30% [41]. If young Japanese females, in general, have a high preoccupation with weight and shape and those with a BMI above 23 kg/m^2^ are particularly concerned regarding their level of body fat, many may restrict their EI to avoid further weight gain or try to hide their actual EI from others. However, it is also important to acknowledge that the BAQ_Salience_ explains EI for only about 10%, suggesting other variables are associated with EI. This suggest interaction with other factors related to EI in this population. One of the possible factors may be response sets such as social desirability and social approval traits [42]. Social desirability has been defined as the defensive tendency of individuals to respond in a manner consistent with societal norms or beliefs whereas social approval has been defined as the tendency for an individual to seek a positive response in testing situations [43]. Previous studies that used a seven-day dietary recall and 24-h recall method found that females with higher social desirability trait under-reported their total energy and fat intake in a seven-day dietary recall relative to a 24-h recall method [43,44]. Seeking a thinner physique may be viewed as one aspect of social desirability and it may be possible that Japanese females, particularly with high salience score (i.e., strong preoccupation toward their body shape and weight), have high social desirability trait and are more likely to under-report their dietary intake. In order to better understand factors that influence EI of young Japanese females, further research is required.

## 5. Conclusions

The present study investigated associations between body image and under-reporting in young Japanese female university students enrolled in nutrition degree. More than 60% of participants were classified as suspected URs based on EI:BMR. As there were no differences in the distribution of grades, nutritional knowledge is not a contributing factor to under-reporting in this population. Rather, the present study indicated associations of body image, particularly an accuracy of understanding current weight and a discrepancy between perceived and ideal weight on EI:BMR and a strong concern and preoccupation on one’s own weight and shape on EI. Considering the fact that young Japanese females are known to have a strong preoccupation toward thinness [6], the results suggest poor validity of dietary assessment outcomes obtained from Japanese females of this particular age group. It may be important for health professionals in Japan to be aware that young Japanese females have a relatively high risk of under-reporting and interpret their results with caution.

It is also important to acknowledge a number of limitations in the present study. Although larger than other studies on nutrition students, the sample size in the present study was relatively small and accordingly, the results may not be generalized. In addition, the present study did not include an objective measure of BMR, essential in the calculation of EI:BMR. We used an equation derived by Ganpule and colleagues [36] which has been reported to be more accurate than other equations for healthy Japanese adults [45], the calculated EI:BMR in the present study may have poor accuracy compared to the value based on measured BMR. Similarly, the present study did not utilize a gold standard method to determine EE such as DLW. Consequently, EB determined from EI and EE in the present study is also likely to be less accurate. In the present study, different methods were used to estimate EE and EER. While EER was estimated from a combination of the BMR equation by Ganpule and colleagues [36] and PAL as suggested for the same age range of Japanese [37], the BAR used an equation specifically derived for this method to determine EE_Record_. In addition, EE_Acc_ from an accelerometer was based on measurement during the daytime and BMR estimated from an equation installed in the device. Differences in methods to estimate EE and EER made it impossible to determine the true value of participants’ EE. Future research is, therefore, recommended to determine EE of participants using a reference method.

## Figures and Tables

**Table 1:** Demographic characteristics of participants (*n* = 100).

Variables	Mean ± SD	Median	Min–Max
Age (years) ^†^	19.1 ± 1.4	19.0	18.0–29.0
Stature (cm)	158.9 ± 5.2	159.2	147.6–171.5
Body mass (kg) ^†^	53.0 ± 6.6	52.2	41.9–88.0
BMI (kg/m^2^) ^†^	21.0 ± 2.3	20.6	17.1–33.8
Σ8SF (mm) ^†^	127.3 ± 38.4	121.0	72.4–293.0
AHtR ^†^	0.47 ± 0.05	0.50	0.40–0.60
WHR ^†^	0.71 ± 0.04	0.70	0.60–0.80
%BF (%) ^†^	28.1 ± 4.5	28.0	16.3–49.2
%TF (%) ^†^	27.2 ± 5.4	26.8	14.6–51.7
Visceral fat level ^†^	2.4 ± 1.7	2.0	1.0–13.0
Perceived health status (%)			
Not good	63.0		
Good	28.0		
Not sure	9.0		
Effort to maintain current weight (%)			
Yes	59.0		
No	41.0		
Past history of weight management (%)			
Yes, multiple times	31.0		
Yes, at least once	36.0		
Never	33.0		
Eat three meals a day (% Yes)	85.0		
Frequency of exercise of more than 30 min per session during a week (% Never)	47.0		

^†^ Variables that did not show normal distribution using the Shapiro–Wilk test. SD: Standard deviation, BMI: Body mass index, Σ8SF: Sum of eight skinfolds, AHtR: Abdominal to height ratio, WHR: Waist to height ratio, %BF: Percentage body fat, %TF: Percentage trunk fat.

**Table 2:** Basal metabolic rate, estimated energy requirement, energy intake from dietary assessment and energy expenditure from physical activity assessments (*n* = 100).

Variables	Mean ± SD	Median	Min–Max
BMR (kcal/day) ^†^	1203 ± 91	1195	1040–1614
EER (kcal/day) ^†^	2105 ± 159	2091	1821–2825
EI (kcal/day)	1523 ± 457	1499	536–2735
Energy contribution from protein (%) ^†^	15.1 ± 3.0	14.9	8.9–30.4
Energy contribution from carbohydrate (%) ^†^	53.9 ± 7.6	54.2	18.9–72.5
Energy contribution from fat (%)	29.3 ± 5.8	29.0	17.0–48.7
EE_Record_ (kcal/day) ^†^	2178 ± 327	2137	1577–3651
EE_Acc_ (kcal/day)	1781 ± 185	1775	1414–2309
Step counts (steps) ^†^	7746 ± 3239	6797	1022–16,602
Diff_EI–EER_ (kcal/day)	−582 ± 496	−633	−1840–815
Diff_EI–EERecord_ (kcal/day)	−655 ± 594	−704	−2836–824
Diff_EI–EEAcc_ (kcal/day)	−258 ± 490	−243	−1259–952
EI:BMR ^†^	1.27 ± 0.4	1.22	0.48–2.52
Percentage of negative EB using EER (%)	87.0		
Percentage negative EB using EE_Record_ (%)	86.0		
Percentage negative EB using EE_Acc_ (%)	75.0		
Percentage EI:BMR < 1.35 (%)	67.0		

^†^ Variables that did not show normal distribution using the Shapiro–Wilk test. SD: Standard deviation, BMR: Basal metabolic rate, EER: Estimated energy requirement, EI: Energy intake, EE_Record_: Energy expenditure from the Bouchard’s physical activity record, EE_Acc_: Energy expenditure from triaxial accelerometer, Diff_EI–EER_: Difference between EI and EER, Diff_EI–EERecord_: Difference between EI and EE_Record_, Diff_EI–EEAcc_: Difference between EI and EE_Acc_, EI:BMR = Energy intake to basal metabolic rate ratio.

**Table 3:** Differences in demographic characteristics between non-under-reporters and suspected under-reporters.

Variables	Under-Reporters (*n* = 67) Mean ± SD	Non-Under-Reporters (*n* = 33) Mean ± SD	*p*-Value
Age (years)	18.9 ± 1.0	19.5 ± 2.0	0.15
Stature (cm)	159.3 ± 5.4	158.1 ± 4.6	0.26
Body mass (kg)	53.9 ± 7.2 *	51.2 ± 4.7	<0.05
BMI (kg/m^2^)	21.3 ± 2.6	20.5 ± 1.7	0.26
Σ8SF (mm)	132.0 ± 42.4	117.8 ± 26.7	0.17
AHtR	0.47 ± 0.05	0.47 ± 0.05	0.89
WHR	0.71 ± 0.04	0.71 ± 0.05	0.78
%BF (%)	28.8 ± 4.8 *	26.7 ± 3.6	0.04
%TF (%)	28.1 ± 5.7 *	25.5 ± 4.3	0.03
Visceral fat level	2.7 ± 1.9 *	1.9 ± 0.9	0.02

SD: Standard deviation, BMI: Body mass index, Σ8SF: Sum of eight skinfolds, AHtR: Abdominal to height ratio, WHR: Waist to height ratio, %BF: Percentage body fat, %TF: Percentage trunk fat. * *p* < 0.05.

**Table 4:** Differences in estimated basal metabolic rate, energy intake and energy expenditure between non-under-reporters and suspected under-reporters.

Variables	Under-Reporters (*n* = 67) Mean ± SD	Non-Under-Reporters (*n* = 33) Mean ± SD	*p*-Value
BMR (kcal/day)	1216 ± 98 *	1176 ± 69	0.03
EER (kcal/day)	2128 ± 171 *	2058 ± 120	0.03
EI (kcal/day)	1280 ± 289 **	2016 ± 314	<0.001
Energy contribution from protein (%)	14.9 ± 3.3	15.5 ± 2.3	0.13
Energy contribution from carbohydrate (%)	54.3 ± 8.3	52.9 ± 5.8	0.23
Energy contribution from fat (%)	29.0 ± 6.4	29.9 ± 4.6	0.29
EE_Record_ (kcal/day)	2226 ± 348 *	2081 ± 257	0.04
EE_Acc_ (kcal/day)	1800 ± 195	1742 ± 158	0.15
Step counts (steps)	7731 ± 3293	7777 ± 3175	0.60
EI:BMR	1.05 ± 0.23 **	1.72 ± 0.30	<0.001

SD: Standard deviation, BMR: Basal metabolic rate, EER: Estimated energy requirement, EI: Energy intake, EE_Record_: Energy expenditure from the Bouchard’s physical activity record, EE_Acc_: Energy expenditure from triaxial accelerometer, EI:BMR: Energy intake to basal metabolic rate ratio. * *p* < 0.05, ** *p* < 0.01.

**Table 5:** Differences in body perception, body dissatisfaction and body concerns between non-under-reporters and suspected under-reporters.

Variables	Under-Reporters (*n* = 67) Mean ± SD	Non-Under-Reporters (*n* = 33) Mean ± SD	*p*-Values
BSS_General_ score	69.4 ±12.6	67.6 ± 11.9	0.48
BSS_Head_ score	32.7 ± 6.9	32.7 ± 7.1	0.96
BSS_Body_ score	36.6 ± 7.7	34.9 ± 7.6	0.39
BAQ_Total_, score	133.9 ± 14.4	127.2 ± 18.3	0.16
BAQ_Fat_, score	46.5 ± 7.8	41.9 ± 10.9	0.05
BAQ_Disparagement_ score	19.5 ± 4.3	18.9 ± 3.7	0.87
BAQ_Strength_ score	17.9 ± 4.0	18.3 ± 3.7	0.59
BAQ_Salience_ score	22.3 ± 3.9	21.2 ± 4.0	0.52
BAQ_Attractive_ score	12.4 ± 2.9	13.2 ± 4.0	0.26
BAQ_Lowerbody_ score	15.2 ± 2.3	13.6 ± 4.0	0.13

SD: Standard deviation, BSS_General_: The BSS “general” body dissatisfaction score, BSS_Head_: The BSS “head” dissatisfaction score, BSS_Body_: The BSS “body” dissatisfaction score, BAQ_Total_: The BAQ total score, BAQ_Fat_: The BAQ feeling fat subscale score, BAQ_Disparagement_: The BAQ disparagement subscale score, BAQ_Strength_: The BAQ strength and fitness subscale score, BAQ_Salience_, The BAQ salience of weight and shape subscale score, BAQ_Attractive_: The BAQ attractiveness subscale score, and BAQ_Lowerbody_: The BAQ Lower body fatness subscale score.

**Table 6:** Regression equations derived from stepwise multiple regression analysis.

Dependent Variables	Equations	R^2^_adj_	SEE
EI:BMR	1.226 + 0.001 × EI − 0.001 × EER + 0.003 × Diff_measured–ideal_ − 0.008 × BSS_Arms_ − 0.007 × BSS_Head_ + 0.007 × Diff_measured–current_ + 0.005 × BSS_Neck_	0.995	0.027
EI	2396.710 − 39.545 × BAQ_Salience_	0.112	426.023

Variables included in analyses were age, grade, stature, body mass, BMI, %BF, %TF, visceral fat level, WHR, AHtR, Σ8SF, BMR, EER, EI, %E_Protein_, %E_CHO_, %E_Fat_, participation to a regular exercise, EE_Record_, EE_Acc_, step counts, perceived current weight, perceived ideal weight for current height, Diff_measured–current_, Diff_measured–ideal_, BSS scores for each body part as well BSS_General_, BSS_Head_, BSS_Body_, BAQ_Total_, BAQ_Fat_, BAQ_Disparagement_, BAQ_Strength_, BAQ_Salience_, BAQ_Attractive_, and BAQ_Lowerbody_. Where EI:BMR: Energy intake to basal metabolic rate ratio, EI: Total energy intake in kcal/day, EER: Estimated energy requirement in kcal/day, Diff_measured–ideal_: Difference between measured body mass and perceived ideal weight in kg, BSS_Arms_: BSS “arms” score, BSS_Head_: BSS “head” score, BSS_Neck_: BSS “neck” score, Diff_measured–current_: Difference between measured body mass and perceived current weight in kg, BAQ_Salience_: The BAQ salience of weight and shape subscale score, R^2^_adj_: Adjusted coefficient of determination, and SEE: Standard error of estimate.
